# Childhood and Maternal Effects on Physical Health Related Quality of Life Five Decades Later: The British 1946 Birth Cohort

**DOI:** 10.1371/journal.pone.0088524

**Published:** 2014-03-26

**Authors:** Gita D. Mishra, Stephanie Black, Mai Stafford, Rachel Cooper, Diana Kuh, for the National Survey of Health and Development scientific and data collection team

**Affiliations:** 1 School of Population Health, University of Queensland, Brisbane, Australia; 2 MRC Unit for Lifelong Health and Ageing, University College London, London, United Kingdom; CUNY, United States of America

## Abstract

Limited research has been done on the relationships between childhood factors and adult physical health related quality of life, with the underlying pathways not fully elucidated. Data from 2292 participants of the British 1946 birth cohort were used to examine the relationship of childhood characteristics and family environment with principal component summary (PCS) scores and the physical functioning (PF) subscale of the SF-36 at age 60–64 years. Impaired physical functioning was defined as the lowest quartile scores in the PF subscale. Childhood factors (father in manual social class versus non-manual (β =  −2.34; 95%CI: −3.39, −1.28) and poor maternal health versus good/excellent maternal health (β =  −6.18; −8.78, −3.57)) were associated with lower PCS scores at 60–64 years. Adult health behaviours (increasing BMI, lifelong smoking, and lower physical activity) at 53 years were identified as strong risk factors for lower PCS scores. After adjusting for these factors and education level (N = 1463), only poor maternal health remained unattenuated (β =  −5.07; −7.62, −2.51). Similarly poor maternal health doubled the risk of reporting impaired PF (Odds ratio =  2.45; 95%CI: 1.39, 4.30); serious illness in childhood (OR = 1.44; 1.01, 2.06) and lower educational level attained were also risk factors for impaired PF (N = 1526). While findings suggest the influence of father's social class on physical health related quality of life are mediated by modifiable adult social factors and health behaviours; health professionals should also be mindful of the inter-generational risk posed by poor maternal health on the physical health related quality of life of her offspring almost five decades later.

## Introduction

With the ageing demographic profile of many nations, research has increasingly turned to understanding the factors that promote and sustain good health-related quality of life – a concept that has been framed as “ageing well” or “healthy ageing”. This shift reflects policy priorities to contain expenditure on health care and to invest in preventive health strategies that support the populace in living healthier for longer [1]. Prevention strategies may need to begin years or even decades before the onset of older age. The maturation of cohort studies that can now draw on data from their participants for health outcomes in later life has also prompted a focus on understanding the role of childhood exposures and intergenerational effects as well as adult factors [2].

Physical health, as indicated by simple objective measures of physical capability such as grip strength, walking speed, and standing balance performance, predict subsequent health outcomes in older populations, including disability and mortality [3]–[5]. A recent systematic review and meta-analysis identified childhood socioeconomic position (SEP) as having modest associations with physical capability levels in adulthood after adjusting for age, for instance those with higher SEP in childhood were more likely to have faster walking speed and chair rise times [6].

Investigations into the impact of SEP over the life course have used health-related quality of life as a reliable subjective measure of health, with the physical component summary (PCS) score of the Short-Form Health Survey (SF-36) providing a validated measure of physical health [7]. The PCS score comprises weighted contributions from all eight subscales, including those for physical functioning (PF), role limitations due to physical problems, bodily pain, and general health. Findings from the Whitehall II study on civil servants in the UK supported the cumulative effect of SEP on PCS scores among men and women, such that greater socioeconomic disadvantage over the life course was associated with increasingly poor physical health [8]. In another study of 9000 employees of the City of Helsinki, childhood circumstances were not directly associated with PCS scores, but were found to act indirectly through adult SEP, with lower education level, occupational class, and income all clearly linked with PCS score [9].

While the PCS score has been adopted as a measure of overall physical health-related quality of life the PF subscale provides a more focused measure of the extent that a person perceives their daily function and activities are impeded by their physical health. Findings from the Helsinki Birth Cohort Study indicate that early life stress increased the risk of impaired PF in men, but not women [10]. Key limitations of these and other similar studies to elucidate pathways, however, has been lack of consideration of childhood factors beyond a few limited indicators of childhood SEP, usually taken at one time point in childhood, and their reliance on retrospective data. In order to investigate the causal pathways involved, further understanding is needed of the relationship of a range of factors in childhood (including family environment, parental health, physical health and personality) on physical health in later life after accounting for potential proximal mediating mechanisms, such as those due to health behaviours.

This study draws on prospective data from the Medical Research Council National Survey of Health and Development (NSHD), a long-running population-based birth cohort study. It investigates the associations of early life factors with physical health and impaired physical functioning at ages 60–64 years and identifies adult risk factors, including adult social class, body mass and physical activity, that may mediate these effects.

## Methods

The NSHD is a birth cohort study consisting of a socially stratified sample of 2,547 women and 2,815 men born during one week in March 1946. There have been 23 follow-ups of the whole cohort, with the most recent sweep at 60–64 years (2006–2011) including physical, cognitive performance assessments, measures of musculoskeletal and cardiovascular systems, and quality of life assessment via postal questionnaire. A total of 2661 responded to the latest sweep (1286 males, 1375 females) giving an overall response rate of 84% [11]. Ethical approval for the study was obtained from the Greater Manchester Local Research Ethics Committee and the Scotland A Research Ethics Committee. All study members provided written informed consent.

### Outcome measures

#### Physical health-related quality of life and impaired physical functioning

At age 60–64 years, the study members completed the SF-36 survey, which is used to measure generic health concepts relevant across age, disease, and treatment groups [12], [13]. For the analysis, higher PCS score denoted better physical health and those in the lowest quartile of the PF subscale were defined as having limited physical functioning.

### Risk factors

As in our previous research [14] factors were chosen as they represented aspects of childhood characteristics and family environment that are thought to be important for health in later life [15].

#### Characteristics of the infant

Birth weight was taken from the medical records around the time of the birth of the child. Information about infant feeding was obtained by interview with the mothers when the cohort members were aged two years and was classified as: mixed or bottle fed, or breast fed only.

#### Family background

Indicators of socioeconomic conditions in childhood, based on information from interviews of the mother at home by health visitors, included crowding in the household at age 2 (< 2 persons per room, ≥ 2 people per room); number of household amenities (running hot water, sole use of a kitchen, and sole use of a bathroom) that were lacking at age 2 (0, 1, 2, 3); type of dwelling tenure (owner occupier, private landlord, council, other); father' social class when the study members were aged 4 years (non-manual or manual).

The mother reported on her own health (excellent, average, poor) and personality, her husband's health (excellent, average, poor) during the home visit when the study member was aged 15. Information on parental divorce, separation and death by that age was obtained from reports near to the occasion throughout the childhood years.

#### Childhood physical health and personality

Physical health during childhood was assessed by maternal reports of lower respiratory infections up until 2 years of age (none, at least one attack) and whether the study member had suffered serious illness that necessitated a hospital admission up until 15 years (none/unknown, yes). Adolescent personality measures, extraversion (−6 to 0, 1 to 3, 4 to 6) and neuroticism (−6 to −2, −1 to 1, 2 to 6) were based on teachers' completion of the short Maudsley Personality Inventory when the study members were aged 15 with higher scores indicating severity of trait.

#### Adult social factors and health behaviours

Educational attainment by 26 years was categorised into no qualifications, below secondary qualifications, ordinary secondary qualifications (‘O’ levels or equivalent), advanced secondary qualifications (‘A’ level or equivalent), and higher qualifications (degree or equivalent). At age 53, household social class was based on the current or most recent occupation of either the study member (for males or unmarried females) or the spouse (for married females), categorised into manual or non-manual groups. Using information on smoking status at ages 20, 26, 31, 36, 43, and 53 years, four categories of lifelong smoking behaviour were defined: never smoker, predominant non smoker, predominant smoker, and lifelong smoker [16]. Physical activity level was measured at age 53 years, based on reports of sports or vigorous leisure activities in the previous month and was grouped as: none, less than once a week, and at least once per week. Body mass index (BMI, kg/m^2^), based on measured height and weight at 53 years according to a standard protocol, was grouped as: underweight (< 20 kg/m^2^); normal weight (≥20 -<25 kg/m^2^); overweight (≥25 -<30 kg/m^2^); and obese (≥30 kg/m^2^).

### Statistical methods

PCS score was calculated by weighting and summing the eight dimensions of SF-36, according to sex-specific weights obtained from a general population sample in the UK [17]. The resulting scores were standardized to a *T*-score, where the mean was set to 50 and standard deviation to 10, with higher scores representing better physical health. The proportions of missing data from each subscale of the SF-36 ranged from 0.5% (for the item “*how is your health in general*”) to 4.5% (*“Does your health now limit you in these activities*: *vigorous activities, such as running…”*). The standard procedure of imputing missing items within a subscale was used, as detailed in the SF-36 manual [18], that is if more than half the items were missing in any given subscale then the value for the entire subscale was set as missing.

The analyses were carried out in 3 steps. The first was to perform univariable regression analysis between the PCS score and the potential risk factors in the four areas: a) characteristics of the infant, b) family environment, c) childhood physical health and personality, and d) adult social factors and health behaviours. All factors that were significant at the 10% level with the PCS score were then subjected to multivariable regression analysis (2^nd^ step) within each of these areas, to identify the factors that remained significant after mutual adjustment. These factors were then selected for the final step to reveal the extent that childhood factors were attenuated or mediated by factors in adult life. This involved: univariable regression for each of the selected variables (Model A), a multivariable regression analysis that incorporated the childhood factors (Model B), and a further multivariable regression analysis (Model C) that included adult characteristics. This approach was repeated using logistic regression analysis to determine the relationships with limited PF. Multiple imputation was also conducted as part of the sensitivity analysis to examine the effects of missing covariate data on the results.

## Results

On average, males recorded slightly higher SF-36 scores for the eight sub-sales (though not statistically different at the individual sub-scale level) than females (Table 1), with their mean PCS score also slightly higher than the UK population norm. Although the univariable regression analysis (Table 2) identified associations across a wide range of factors in childhood, including higher level of crowding at age 2 years and respiratory illness in early life, no evidence was found to relate early life nutrition (i.e. birth weight and breast feeding) with physical health at age 60–64 years. Similarly no association was found for parental divorce, paternal or maternal death by age 15, or extroversion at age 15. All of the social factors and health behaviours in adulthood considered here were associated with the PCS score. Further analysis allowing for mutual adjustment in each area (family environment, childhood physical health and personality, and adult social factors and health behaviours) resulted in selection of just four childhood factors for use in subsequent analysis: father's social class at 4 years, ‘mother’s health summary at 15 years, serious illness (0–15 years), and neuroticism at 15 years. All adult social factors and health behaviours remained significant, except for head of house social class at 53 years (results not shown).

**Table 1:** Mean (SD) scores for the eight dimensions and the two summary measures of SF-36 for males and females aged 60–64 years.

		All		Males		Females	
		N	Mean (SD)	N	Mean (SD)	N	Mean (SD)
**Dimensions**	Physical Functioning	2422	81.1 (23.8)	1164	83.9 (23.2)	1258	78.5 (24.1)
	Role Physical	2400	81.0 (34.9)	1162	83.6 (33.1)	1238	78.5 (36.3)
	Bodily Pain	2434	74.5 (25.4)	1170	76.9 (24.7)	1264	72.2 (25.8)
	General Health	2384	71.5 (20.6)	1146	70.9 (20.6)	1238	72.0 (20.5)
	Vitality	2438	64.6 (19.7)	1172	66.2 (19.1)	1266	63.2 (20.2)
	Social Functioning	2453	87.4 (22.1)	1180	88.5 (21.6)	1273	86.4 (22.5)
	Role Emotion	2383	89.7 (27.0)	1153	90.7 (25.9)	1230	88.9 (27.9)
	Mental Health	2438	78.6 (16.3)	1172	79.8 (16.2)	1266	77.5 (16.3)
**Summary scores**	Physical component summary score	2292	50.7 (10.4)	1110	51.1 (10.1)	1182	50.4 (10.7)

**Table 2:** Factors across the life course and physical component summary scores from the Short Form-36 at age 60–64 years.

				Physical Component Summary score	
			N (%)	Unadjusted beta coefficient (95% CI)	*P* *
*Characteristics of the infant*	Birth weight sex-standardised		2287	Reference −0.30 (−0.73,0.12)	0.2
	**Infant feeding**				0.8
		Mixed or bottle fed	1479 (69%)	Reference	
		Breast fed only	650 (31%)	−0.11 (−1.07, 0.85)	
*Family background*	**Household crowding at 2 years**				<0.0001
		< 2 persons per room	1763 (81%)	Reference	
		≥2 people per room	413 (19%)	−2.19 (−3.30, −1.08)	
	**Number of home amenities lacking at 2 years**				0.004
		0	1055 (50%)	Reference	
		1	375 (18%)	−0.88 (−2.10, 0.33)	
		2	576 (27%)	−1.96 (−3.01, −0.91)	
		3	93 (4%)	−0.71 (−2.90, 1.49)	
	**Home ownership at 4 years**				0.002
		Owner occupier	638 (29%)	Reference	
		Private landlord	837 (39%)	−1.04 (−2.11, 0.02)	
		Council	586 (27%)	−2.08 (−3.24, −0.92)	
		Other	105 (5%)	−2.64 (−4.78, −0.50)	
	**Father's occupational social class at 4 years**				<0.0001
		Non-manual	974 (46%)	Reference	
		Manual	1148 (54%)	−2.29 (−3.17, −1.41)	
	**Mother's health summary at 15 years**				<0.0001
		Excellent	1394 (71%)	Reference	
		Average	493 (25%)	−2.77 (−3.83, −1.70)	
		Poor	78 (4%)	−5.61 (−7.97, −3.25)	
	**Father's health summary at 15 years**				<0.0001
		Excellent	1441 (74)	Reference	
		Average	397 (21)	−1.77 (−2.93, −0.61)	
		Poor	98 (5%)	−4.43 (−6.57, −2.29)	
	**Mother's neuroticism at 15 years**				0.02
		0 symptoms	668 (33.8)	Reference	
		1	449 (22.7)	0.21 (−1.04, 1.46)	
		2	389 (19.7)	−0.09 (−1.40, 1.21)	
		3	217 (11.0)	0.69 (−0.91, 2.29)	
		4–6	252 (12.8)	−2.26 (−3.78, −0.75)	
	**Parental divorce by 15 years**		2292		0.5
		No		Reference	
		Yes		−0.73 (−2.68, 1.23)	
	**Father's death by 15 years**		2057		0.6
		No		Reference	
		Yes		−0.52 (−2.58, 1.54)	
	**Mother's death by 15 years**		2084		0.3
		No		reference	
		Yes		−1.61 (−4.62, 1.39)	
*Childhood physical health and personality*	**Lower respiratory infection (0**–**2 years)**				0.004
		None	1621 (76%)	Reference	
		At least one attack	518 (24%)	−1.51 (−2.52, −0.49)	
	**Serious illness (0–15 years)**				0.001
		None/Unknown	1957 (85%)	Reference	
		Yes	335 (15%)	−2.10 (−3.30, −0.90)	
	**Extroversion at 15 years**				0.27
		−6 to 0 (least extrovert)	620 (32%)	Reference	
		1 to 3	644 (34%)	−0.44 (−1.58, 0.71)	
		4 to 6 (most extrovert)	654 (34%)	0.50 (−0.64, 1.64)	
	**Neuroticism at 15 years**				0.0004
		−6 to −2 (least neurotic)	771 (40%)	Reference	
		−1 to 1	361 (19%)	−2.41 (−3.70, −1.12)	
		2 to 6 (most neurotic)	806 (42%)	−1.48 (−2.49, −0.46)	
*Adult social factors and health behaviours*	**Highest education qualification achieved by 26 years**				<0.0001
		No qualifications	742 (34%)	Reference	
		Ordinary level	573 (26%)	1.52 (0.38, 2.65)	
		Advanced level	599 (28%)	2.40 (1.28, 3.52)	
		Degree level or above	255 (12%)	4.46 (2.98, 5.94)	
	**Head of house social class at 53 years**				<0.001
		Non-manual	1406 (67%)	Reference	
		Manual	695 (33.1)	−1.92 (−2.83, −1.00)	
	**Body Mass Index at age 53 (kg/m^2^)**				<0.0001
		< 20	39 (1.8%)	−2.59 (−5.86, 0.67)	
		20 ≤ BMI <25	669 (31%)	Reference	
		25 ≤ BMI < 30	936 (44%)	−1.42 (−2.42, −0.42)	
		BMI ≥ 30	485 (23%)	−5.71 (−6.89, −4.52)	
	**Physical activity per week at 53 years**				<0.0001
		None	954 (45%)	Reference	
		Less than once/week	415 (19%)	2.64 (1.47, 3.81)	
		More than once/week	772 (36%)	3.81 (2.85, 4.78)	
	**Lifetime smoking trajectory to 53 years**				<0.0001
		Never smoker	677 (32%)	Reference	
		Predominantly non-smoker	758 (35%)	−0.11 (−1.18, 0.95)	
		Predominantly smoker	437 (20%)	−2.62 (−3.86, −1.39)	
		Lifelong smoker	276 (13%)	−4.91 (−6.35, −3.47)	

*P-values from ANOVA.

Compared with the univariable regression analysis (Table 3, Model A) for the selected factors and for those study members with complete covariate data, the results for childhood physical health and personality (Table 3, Model B) were attenuated after adjusting for family background. The full model (Table 3, Model C), which includes adult social factors and health behaviours, indicates that higher levels of BMI, lower physical activity, and lifetime smoking remained strongly associated with lower PCS scores in later life, whereas the positive association of level of education was attenuated in the full model. For instance, the effect of being obese at age 53 or a lifelong smoker was to lower the normalised PCS score by more than 4 units, or nearly half a standard deviation. In contrast, among the family background factors only the influence of mother's health when the participant was aged 15 remained as having a strong additional association with a lower PCS score in later life, specifically poor maternal health was associated with PCS score more than half a standard deviation lower (−5.07 units) compared with when the mothers reported excellent maternal health. The influence of father's social class was completely attenuated in the full model.

**Table 3:** Factors across the life course and health related quality of life – physical component summary measure (PCS) (N = 1463).

			Model A		Model B		Model C	
	Exposure		Beta coefficient (95% CI)	*P*	Beta coefficient (95% CI)	*P*	Beta coefficient (95% CI)	*P*
*Family background*	**Father's occupational social class at 4 years**	Non-manual	Reference	<0.0001	Reference	<0.0001	Reference	0.32
		Manual	−2.34 (−3.39, −1.28)		−1.92 (−2.97, −0.86)		−0.58 (−1.71, 0.56)	
	**Mother's health summary at 15 years**	Excellent	Reference	<0.0001	Reference	<0.0001	Reference	<0.0001
		Average	−2.83 (−4.06, −1.61)		−2.37 (−3.61, −1.14)		−2.17 (−3.38, −0.97)	
		Poor	−6.18 (−8.78, −3.57)		−5.70 (−8.30, −3.10)		−5.07 (−7.62, −2.51)	
*Childhood physical health and personality*	**Serious illness (0–15 years)**	None	Reference	0.05	Reference	0.098	Reference	0.12
		Yes	−1.47 (−2.95, 0.01)		−1.23 (−2.69, 0.23)		−1.14 (−2.57, 0.28)	
	**Lower respiratory infection (0–2 years)**	None	Reference	0.08	Reference	0.49	Reference	0.70
		At least one attack	−1.11 (−2.34, 0.13)		−0.43 (−1.66, 0.80)		0.24 (−0.96, 1.44)	
	**Neuroticism at 15 years**							
	**Neuroticism at 15 years**	−6 to −2 (least neurotic)	Reference	0.02	Reference	0.06	Reference	0.14
		−1 to 1	−1.97 (−3.46, −0.49)		−1.60 (−3.07, −0.14)		−1.43 (−2.86, −0.0008)	
		2 to 6 (most neurotic)	−1.18 (−2.35, −0.02)		−1.06 (−2.21, 0.10)		−0.62 (−1.76, 0.52)	
*Adult social factors and health behaviours*	**Highest education qualification achieved by 26 years**	No qualifications	Reference	<0.0001			Reference	0.27
		Ordinary level	1.82 (0.47, 3.16)				0.42 (−0.94, 1.77)	
		Advanced level	2.61 (1.26, 3.97)				0.64 (−0.79, 2.06)	
		Degree level or above	5.14 (3.35, 6.93)				1.92 (0.004, 3.85)	
	**Body Mass Index (kg/m^2^) at age 53 years**	< 20	Reference	<0.0001			Reference	<0.0001
		≥20 −<25	−1.54 (−5.41, 2.34)				−0.51 (−4.30, 3.27)	
		≥25 −<30	−1.54 (−2.8, −0.33)				−1.37 (−2.55, −0.19)	
		≥ 30	−4.93(−6.38, −3.49)				−4.31 (−5.73, −2.90)	
	**Physical activity per week at 53 years**	None	Reference	0.00001			Reference	0.005
		Less than once/week	2.09 (0.68, 3.49)				0.92 (−0.46, 2.30)	
		More than once/week	3.27 (2.09, 4.44)				1.95 (0.78, 3.12)	
	**Lifetime smoking trajectory to 53 years**	Never smoker	Reference	<0.0001			Reference	<0.0001
		Predominantly non-smoker	0.33 (−0.93, 1.60)				0.12 (−1.11, 1.36)	
		Predominantly smoker	−2.29 (−3.76, −0.82)				−1.87 (−3.32, −0.42)	
		Lifelong smoker	−5.35 (−7.06, −3.64)				−4.42 (−6.13, −2.71)	

Model A: univariable regression analysis for those with complete covariate data; Model B multivariable regression: includes childhood psychosocial factors; and personality; Model C: includes all factors in Model B and adult social factors and health behaviours.

A similar pattern was evident for limited PF (Table 4), with a clear relationship for adult health behaviours: obese cohort members (BMI ≥ 30) had three times the odds of limited PF than those who were underweight (OR: 3.04, 95% CI: 2.11–4.38), while lifelong smokers had more than double the odds of lifelong non-smokers (2.40, 1.61–3.57). Those who engaged in physical activity at least once per week had half the odds of reporting limited PF when compared with those who were inactive (0.44, 0.32–0.61), while education level remained as having some protective effect. In terms of childhood factors father's social class and neuroticism at 15 years were no longer statistically significant in the full model. The association of maternal health when the study member was aged 15 continued to indicate additional risk for limited PF; those whose mother reported poor health were more than twice as likely (2.72, 1.52–4.85) to report limited PF in later life compared with the reference group whose mothers reported excellent health. After adjusting for all the other factors, those who had a serious illness in childhood were also at increased risk of reporting limited PF at 60–64 years.

**Table 4:** Factors across the life course and limited physical functioning (PF) from SF-36 (N =  1526).

			Model A		Model B		Model C	
			Odds ratio (95% CI)	*P*	Odds ratio (95% CI)	*P*	Odds ratio (95% CI)	*P*
*Family background*	**Father's occupational social class at 4 years**	Non-manual	Reference	<0.0001	Reference	<0.0001	Reference	0.12
		Manual	2.05 (1.58, 2.66)		1.92 (1.48, 2.51)		1.27 (0.94, 1.71)	
	**Mother's health summary at 15 years**	Excellent	Reference	<0.0001	Reference	0.0001	Reference	0.003
		Average	1.65 (1.26, 2.18)		1.44 (1.09, 1.91)		1.36 (1.00, 1.83)	
		Poor	3.19 (1.9, 5.34)		2.80(1.65, 4.75)		2.45 (1.39, 4.30)	
	**Lower respiratory infection (0–2 years)**	None	Reference	0.02	Reference	0.17	Reference	0.99
		At least 1 attack	1.38 (1.05, 1.82)		1.22 (0.92, 1.62)		1.00 (0.74, 1.35)	
*Childhood physical health and personality*	**Serious illness (0–15 years)**	None	Reference	0.02	Reference	0.04	Reference	0.04
		Yes	1.46 (1.06, 2.02)		1.41 (1.01, 1.97)		1.44 (1.01, 2.06)	
	**Neuroticism at 15 years**	−6 to −2 (least neurotic)	Reference	0.02	Reference	0.07	Reference	0.1
		−1 to 1	1.62 (1.16, 2.26)		1.49 (1.06, 2.10)		1.45 (1.01, 2.08)	
		2 to 6 (most neurotic)	1.18 (0.89, 1.56)		1.15 (0.87, 1.53)		1.04 (0.77, 1.41)	
*Adult social factors and health behaviours*	**Highest education qualification achieved by 26 years**	No qualifications	Reference	<0.0001			Reference	0.02
		Ordinary level	0.53 (0.39, 0.71)				0.75 (0.54, 1.05)	
		Advanced level	0.39 (0.28, 0.54)				0.63 (0.44, 0.91)	
		Degree level or above	0.19 (0.10, 0.33)				0.43 (0.23, 0.81)	
	**Body Mass Index (kg/m^2^)**	< 20	Reference	<0.0001			Reference	<0.0001
		≥20 -<25	1.31 (0.48, 3.55)				0.98 (0.34, 2.79)	
		≥25 -<30	1.56 (1.13, 2.16)				1.49 (1.06, 2.09)	
		≥ 30	3.36 (2.39, 4.73)				3.04 (2.11, 4.38)	
	**Physical activity per week at 53 years**	None	Reference	<0.0001			Reference	<0.0001
		Less than once per week	0.46 (0.33, 0.65)				0.59 (0.41, 0.84)	
		At least once per week	0.34 (0.25, 0.46)				0.44 (0.32, 0.61)	
	**Lifetime smoking trajectory to 53 years**	Never smoker	Reference	<0.0001			Reference	<0.0001
		Predominantly non-smoker	0.88 (0.63, 1.23)				0.90 (0.63, 1.28)	
		Predominantly smoker	1.80 (1.28, 2.54)				1.68 (1.16, 2.43)	
		Lifelong smoker	3.10 (2.15, 4.46)				2.40 (1.61, 3.57)	

Model A: univariable regression analysis; Model B: includes childhood psychosocial factors and personality; Model C: includes all factors in Model B and adult social factors and health behaviours.

## Discussion

As far as we are aware this is the first population based study to use prospective data to examine the influence of a wide range of childhood factors (including infant characteristics, parental health and family background, and childhood physical health and personality) and adult social factors and health behaviours on physical health and limited PF as measured by SF-36 five decades later. The findings clearly show the impact of established modifiable risk factors in adulthood (BMI, smoking, and physical activity) for physical health and limited PF. Some key early life factors, however, remained associated: maternal health when the participant was aged 15 was a strong additional risk factor for physical health. Serious illness during childhood was also associated with an additional risk of limited PF in later life, while higher education level had some additional beneficial effects.

Findings from this study highlight the mediating role of adult health behaviours and are consistent with a systematic review and meta-analysis of factors affecting objective measures of physical capability [6]. Specifically, adjustment for adult SEP and contemporaneous body size (height, weight, and BMI) greatly attenuated associations of lower childhood SEP with physical capability in later life. The findings are also consistent with previous analyses of the associations of indicators of SEP across life with objective measures of physical capability at age 53 years in the NSHD [19]. These analyses showed links between lower levels of both maternal education and father's occupational class and poorer performance in chair-rising and standing-balance tests that were attenuated after adjustment for indicators of childhood growth, neurodevelopment, home environment, and indicators of adult SEP and health behaviours.

Proximal adult health behaviours (identified in this study by BMI, physical activity, and lifelong smoking) may nevertheless be initiated in childhood. A recent review has highlighted evidence from several life course studies on the influence of childhood socioeconomic position and the trajectory of BMI across the life course, with less advantaged childhood SEP linked with a more rapid gain in BMI at different life stages [2]. Smoking behavior provides another example, with lifelong smoking in this study based on reports spanning from age 20 to 53 years. Previous findings from the NSHD have shown that a third of the lifelong smokers initiated their smoking behavior before the age of 16 [16].

The relationship of early child health and development with the developmental trajectory through adolescence and on subsequent adult outcomes, such as educational attainment, has been studied [15]. Apart from biologic and genetic effects, these are increasingly understood in terms of an interplay of factors that describe the nurturing qualities of the family and social environment where children grow up, such as the effect of maternal mental and physical health [15]. Previous research has also focused on the maternal influence with respect to very early life, especially the intrauterine environment. One of the few studies to examine physical health (as obtained from the quality of life instrument) of the child (at age 13 years) found associations with maternal health in pregnancy and maternal rating of overall child health at 5 years [20]. Findings from our study underscore the enduring impact of poor maternal health, after accounting for adult lifestyle and socioeconomic factors on both physical health related quality of life and physical functioning some five decades later. Even though this factor pertained to when the participant was aged 15, reporting of poor maternal health may reflect an on-going rather than transient condition that characterised the home environment. These findings are also consistent with previous research from the NSHD that has found both the experience of parental illness was strongly linked with unexplained symptoms in childhood, such as abdominal pain, and that unexplained symptoms in childhood were associated with symptoms in adulthood [21].

The lack of evidence for an association of birthweight, where this has previously been associated with grip strength in the NSHD, may reflect the difference between the objective measures used previously, and SF-36 scales that measure the *perceived* physical health-related quality of life and impact of poor physical health functioning in daily life.

For policymakers and health professionals, the findings underline not only the intergenerational and long-term effects when mothers and children have poor health, but also the link between childhood social factors and mediating health behaviours in adulthood. In no way do they lessen, however, the role that preventive health measures can have to modify poor adult health behaviour and improve physical health in older age.

Although the cohort in this study grew up in a period of social change of post-war Britain, it seems likely that the role of maternal health as an additional risk factor for adult physical health remains in subsequent generations. It may be the case, however, that changing family structures – such as single parent families – and changes in the women's societal role may result in the influence of other factors, so that maternal SEP rather than just paternal SEP are needed to characterise childhood circumstances. Further research is needed to determine if the relationships found in this study are evident in different populations and subsequent cohorts, and investigate the detailed pathways across life that provide optimal outcomes for healthy ageing.

### Strengths and limitations

This study draws on data gathered from a large representative birth cohort, with longitudinal data spanning 65 years with the last data collection achieving an 84% response rate [11]. The proportions of missing items were low and comparable to other UK based population studies [22]. Previous findings indicate that the social class and unemployment profiles of on-going NSHD participants is similar to the 2001 England Census reference population, though with higher rates of home ownership and lower prevalence of limiting illness [11]. The use of prospective data on childhood factors also avoids the issues associated with retrospective data collection commonplace in many studies, especially recall bias. Results from analysis of participants with complete covariate data were presented here, as the use of multiple imputation to account for the effects of missing covariate data showed no substantive difference in results.

PCS scores derived from SF-36 are also subject to on-going debate regarding the appropriate method of calculation (using orthogonal versus oblique factor analysis), though the issue of variation in summary scores due to the two methods mainly concerns the measurement of change in quality of life over time and mental health in older age groups (>70 years) [23]. Some caution is also needed in interpreting associations for factors in adulthood due to bi-directional effects, with poor physical health potentially leading to lower physical activity and/or higher BMI, however this issue is minimised in this study as data for these factors were collected at age 53 nearly a decade prior to the SF-36 survey and the analyses were also adjusted for the presence of serious childhood illness.

## Conclusions

Poor maternal health poses risks for physical health related quality of life of the child much later in life. The influence of childhood SEP, as indicated by father's occupational class, was mediated by adult health behaviors. Policymakers and health professionals need to consider these inter-generational and enduring effects as part of preventive health strategies for promoting healthy ageing over the long term.
